# Characterization of Non-Ischemic Dilated Cardiomyopathy in a Native Tanzanian Cohort: MOYO Study

**DOI:** 10.5334/gh.1298

**Published:** 2024-02-29

**Authors:** Lulu Said Fundikira, Pilly Chillo, Mohamed Z. Alimohamed, Henry Mayala, Engerasiya Kifai, Geofrey M. Aloyce, Appolinary Kamuhabwa, Gideon Kwesigabo, Linda W. van Laake, Folkert W. Asselbergs

**Affiliations:** 1 Muhimbili University of Health and Allied Sciences, Dar es Salaam, United Republic of Tanzania, Tanzania; 2 Jakaya Kikwete Cardiac Institute, Dar es Salaam, Tanzania; 3 Tanzania Human Genetics Organization, Dar es Salaam, Tanzania; 4 Department of Cardiology, Division of Heart and Lungs, University Medical Centre Utrecht, Utrecht University, Utrecht, The Netherlands; 5 Amsterdam University Medical Centers, Department of Cardiology, University of Amsterdam, Amsterdam, The Netherlands; 6 Health Data Research UK and Institute of Health Informatics, University College London, London, United Kingdom; 7 The National Institute for Health Research University College London Hospitals Biomedical Research Centre, University College London, London, United Kingdom

**Keywords:** non-ischemic dilated cardiomyopathy, heart failure, echocardiography

## Abstract

**Background:**

Non-ischemic dilated cardiomyopathy (NIDCM) is a common cause of heart failure with progressive tendency. The disease occurs in one in every 2,500 individuals in the developed world, with high morbidity and mortality. However, detailed data on the role of NIDCM in heart failure in Tanzania is lacking.

**Aim:**

To characterize NIDCM in a Tanzanian cohort with respect to demographics, clinical profile, imaging findings and management.

**Methods:**

Characterization of non-ischemic dilated cardioMyOpathY in a native Tanzanian cOhort (MOYO) is a prospective cohort study of NIDCM patients seen at the Jakaya Kikwete Cardiac Institute. Patients aged ≥18 years with a clinical diagnosis of heart failure, an ejection fraction of ≤45% on echocardiography and no evidence of ischemia were enrolled. Clinical data, echocardiography, electrocardiography (ECG), coronary angiography and stress ECG information were collected from February 2020 to March 2022.

**Results:**

Of 402 patients, n = 220 (54.7%) were males with a median (IQR) age of 55.0 (41.0, 66.0) years. Causes of NIDCM were presumably hypertensive n = 218 (54.2%), idiopathic n = 116 (28.9%), PPCM n = 45 (11.2%), alcoholic n = 10 (2.5%) and other causes n = 13 (3.2%). The most common presenting symptoms were dyspnea n = 342 (85.1%), with the majority of patients presenting with New York Heart Association (NYHA) Class III n = 195 (48.5%). The mean (SD) left ventricular ejection fraction (LVEF) was 29.4% (±7.7), and severe systolic dysfunction (LVEF <30%) was common n = 208 (51.7%). Compared with other forms of DCM, idiopathic DCM patients were significantly younger, had more advanced NYHA class (p < 0.001) and presented more often with left bundle branch block on ECG (p = 0.0042). There was suboptimal use of novel guidelines recommended medications ARNI n = 10 (2.5%) and SGLT2 2-inhibitors n = 2 (0.5%).

**Conclusions:**

In our Tanzanian cohort, the majority of patients with NIDCM have an identified underlying cause, and they present at late stages of the disease. Patients with idiopathic DCM are younger with more severe disease compared to other forms of NIDCM.

## Introduction

Non-ischemic dilated cardiomyopathy (NIDCM) is characterized by contractile dysfunction and left ventricular dilatation in the absence of significant coronary artery disease or valvular disease. Non-ischemic dilated cardiomyopathy is a common cause of heart failure with progressive tendency and poor outcome [1]. It is reported to occur in one in 2,500 individuals in the general population of developed countries and has been shown to have high morbidity and mortality [2,3]. Contrary to high-income countries where ischemic heart disease constitutes the major cause of cardiovascular mortality, in Africa, non-ischemic causes such as hypertension with its complications are currently the leading cause of heart failure [4,5]. A systematic review involving sub-Saharan Africa (SSA) countries reports that hypertension is a key modifiable risk factor in heart failure in general as well as in cardiomyopathies [6]. There is a higher prevalence of hypertension in SSA compared to high-income countries [5].

Non-ischemic dilated cardiomyopathy can be of primary origin (genetic, mixed, or predominantly familial non-genetic) or acquired or secondary (e.g., hypertensive, infiltrative or autoimmune) [7]. Apart from genetic causes, there are other recognized specific forms of NIDCM, such as alcohol-induced, peripartum, and HIV-induced DCM, although some genetic mutations may influence their occurrence [8,9]. Putting Tanzania into context, various studies have been conducted showing a tendency for a surge in ischemic cardiomyopathy, as seen elsewhere in the continent [4]. Notwithstanding, various etiologies leading to NIDCM constitute major causes of cardiovascular morbidity and mortality [2]. Although the Tanzania Heart Failure study (TaHeF) involving 427 patients did not report directly on NIDCM, it recorded only 9% of the study population in heart failure due to ischemic cardiomyopathy, suggesting that the majority fell into the non-ischemic category [10]. A recent study done in Tanzania showed echocardiographic etiologies of heart failure (n = 459) were hypertension 40.1%, dilated cardiomyopathy 27%, rheumatic heart disease 23.2% and others 9.3% [11].

There is a dearth of information regarding the demographics, clinical profile, and imaging findings of NIDCM in the Tanzanian setup. The heterogeneous nature of the condition, with its diverse etiologies, calls for a detailed study of the Tanzanian cohort consisting mainly of black ethnicity to identify any peculiar characteristics. Currently, the available management guidelines for NIDCM are based on predominantly European white populations [12].

Characterization of non-ischemic dilated cardioMyOpathY in a native Tanzanian cOhort (MOYO), which literally translates to ‘heart’ in Swahili, aims to characterize NIDCM in terms of demographics, clinical profile, imaging as well and management offered in a Tanzanian cohort. This information may help in creating awareness and guidance in preventive measures as well as early detection of patients at risk to effectuate timely management.

## Methods

### Study design and area

This is a prospective cohort study to characterize NIDCM at Jakaya Kikwete Cardiac Institute (JKCI). Jakaya Kikwete Cardiac Institute is the only tertiary-level hospital and the topmost referral hospital for cardiac diseases in Tanzania, which has a population of 64 million people, receiving cardiac patients from all regions of Tanzania. It has a bed capacity of 104 and around 1,600–2,000 outpatient weekly load. It also has an echocardiography laboratory that has seven ultrasound machines performing around 350 echocardiograms weekly. It also has two catheterization laboratories with 40 procedures per week, of which the majority are coronary angiography (n = 30) and percutaneous cardiac intervention (n = 10).

### Study population, inclusion, and exclusion criteria

All patients aged 18 and above with clinical diagnosis of heart failure and echocardiographic diagnosis of NIDCM with ejection fraction ≤45% at recruitment were enrolled. Exclusion criteria were signs of ischemic cardiomyopathy or valve disease on echocardiography, ECG, exercise stress testing or coronary angiography [13]. Data was collected from echocardiography lab, catheterization lab, inpatient wards and outpatient clinics from February 2020 to March 2022. We had 409 eligible candidates for the study. However, three patients did not consent, and four patients did not have complete echocardiography reports; these seven patients were not involved in the final analysis. We present data from 402 patients. All patients underwent echocardiography and electrocardiography. However, an exercise stress test was done on n = 17, and coronary angiography was done on n = 36 patients.

### Data collection and definition of terms

#### Questionnaire

A structured questionnaire was used to record patients’ socio-demographic and clinical data, including age, sex, hypertension and diabetes history, cigarette smoking and alcohol intake. Other information, including duration of heart failure symptoms, obstetric history, family history of similar illness, and sudden cardiac death, was collected. For each patient, a detailed history of the presenting symptoms was documented.

Dyspnea severity was used to grade patients into different classes of New York Heart Association (NYHA) classes I–IV according to their symptoms, depending on the degree of effort needed to elicit symptoms [12]. Non-ischemic dilated cardiomyopathy, presumably due to Hypertension, was diagnosed as the end stage of hypertensive heart disease, usually the result of longstanding pressure overload, and consists of dilated cardiomyopathy with both diastolic dysfunction and reduced ejection fraction [14]. Peripartum cardiomyopathy (PPCM) was defined as the onset of cardiomyopathy during the last month of pregnancy or within five months of delivery [15]. Alcohol abuse was defined as the consumption of more than 14 units of alcohol in one week. Alcoholic cardiomyopathy was considered present when the cardiomyopathy was detected in an individual with a significant history of alcohol abuse in the absence of other known causes of myocardial disease [16]. In this study, patients were grouped as DCM of other causes if they had underlying causes such as infection (e.g. HIV) or hormonal or drug-induced disease. Familial DCM was identified in patients with idiopathic DCM when two or more individuals in first-degree or second-degree relatives have/had DCM, or the presence of index patient with DCM and a first-degree relative with autopsy-proven DCM or sudden death at ≤50 years of age [17].

#### Laboratory investigations

Routine laboratory test results of hemoglobin level and serum electrolytes were collected. According to the World Health Organization (WHO), anemia is defined as hemoglobin (Hb) levels <12.0 g/dL in women and <13.0 g/dL in men [18]. Serum sodium is defined as normo-natremia (serum Na^+^ 135 to 145 mEq/l, hyponatremia (serum Na^+^ <135 mEq/l) and hypernatremia (serum Na^+^ > 145 mEq/l).

#### ECG and exercise stress testing

A 12-lead resting electrocardiogram was obtained from all patients. A GEMAC2000 machine was used. An experienced cardiologist did the reading and interpretation of the ECG. Some patients underwent exercise stress testing using General Electric CASE V6.73 stress system applying the Bruce protocol; machine printouts were reviewed and confirmed by an experienced cardiologist [19]. Ischemia was considered absent when there was no evidence of unequivocal pathological Q waves and/or ST-segment elevation or depression in serial recordings [20].

#### Cardiac imaging

Chest radiographs obtained from routine care were interpreted by the principal investigator, who is an experienced radiologist, noting the cardiothoracic ratio, cephalization and evidence of pulmonary edema and pleural effusion. Cardiomegaly in chest radiographs is considered present when the thoracic horizontal width of the heart divided by the widest internal diameter of the thorax (i.e. the cardio-thoracic ratio) is above 0.5 [21].

Echocardiography was performed on all patients, recording information on chamber sizes, ventricular systolic function, valvular regurgitation, presence of thrombi, regional myocardial function and pericardial effusion. Left ventricular ejection fraction (LVEF) was evaluated using the biplane method of disks (modified Simpson’s) as per the American Society of Echocardiography and the European Association of Cardiovascular Imaging guidelines [22]. Left ventricular systolic dysfunction was graded as mild (LVEF 41–51%) in males and (41–53%) in females, moderate (LVEF 30–40%), or severe dysfunction (LVEF <30%) [22]. Visual assessment of the left ventricular ejection fraction was done as per the literature [23]. Right ventricular systolic function was evaluated using tricuspid annular plane systolic excursion (TAPSE), with TAPSE <17 considered as right ventricular systolic dysfunction. Myocardial infarction and ischemia were assessed using the 17-segment model in which regional motion for each segment was analyzed individually in multiple views. The scoring used was normal or hyperkinetic, hypokinetic, akinetic and dyskinetic [22].

#### Medications

Information on the patients’ current medication use was collected from the hospital’s electronic Health Information Management System (HIMS) as well as elicited during interviews with patients.

### Data entry and analysis

Data was entered and organized in the REDCap® account hosted at the Muhimbili University of Health and Allied Sciences (MUHAS) [24]. The data were analyzed using IBM SPSS Statistics for Windows (Version 26.0, IBM Corp, Armonk, NY). Descriptive statistics were used to describe the socio-demographic and clinical characteristics of the study participants. We used frequencies for categorical variables and median (IQR) for numerical variables, as the numerical variables were not normally distributed. Comparisons of characteristics between participants with different causes of cardiomyopathy were carried out using the chi-square test for categorical variables and the Mann-Whitney test for numerical variables. Any variable that showed a difference in occurrence between participants with idiopathic cardiomyopathy versus other causes of cardiomyopathy with p < 0.05 was considered statistically significant.

### Ethical considerations

This study was conducted in accordance with the Helsinki Declaration of Studies on Human Subjects. The study was approved by the Directorate of Research and Publications of MUHAS (P. MUHAS – REC-9-2019-060). Participation in the study was voluntary and all patients were educated about the importance of the study. Those who agreed had to sign informed consent forms before any data was collected. Patients who refrained from participation were assured of unbiased medical care. Patients’ data were coded to ensure privacy and confidentiality.

## Results

A total of 402 patients had a clinical and echocardiographic diagnosis of DCM; a slight majority were males (n = 220, 54.7%). The median (IQR) age was 55 (41.0, 66.0). Hypertension, obesity, alcohol consumption, diabetes mellitus and smoking were found in n = 219 (54.2%), n = 95 (23.6%), n = 71 (15.9%), n = 52 (12.9%) and n = 32 (8.0%) respectively. Reported familial disease was recorded in 12 (10.6%) of DCM patients, Table 1.

**Table 1:** Socio-demographic and clinical characteristics of patients with NIDCM in native Tanzanian Cohort: MOYO Study.


VARIABLE	FREQUENCY (%)/MEDIAN (IQR)

Median (IQR) age (years)	55.0 (41.0, 66.0)

Age groups (years), n (%)	

18–30	44 (10.9)

31–45	82 (20.4)

46–55	81 (20.1)

>55	195 (48.5)

Male sex, n (%)	220 (54.7)

Marital status, n (%)	

Single/widowed/separated	92 (22.9)

Married/cohabitating	310 (77.1)

Level of education, n (%)	

No formal education	28 (7.0)

Primary	238 (59.2)

Secondary	98 (24.4)

College/University	38 (9.5)

Insured patients, n (%)	214 (53.2)

Smoking, n (%)	32 (8.0)

Alcohol consumption, n (%) Alcohol abuse, n (%)	71 (17.7) 12(3.0)

Median (IQR) Systolic Blood Pressure (mmHg)	125 (109, 136)

Median (IQR) Diastolic Blood Pressure (mmHg)	80 (71, 90)

Median (IQR) Body Mass Index (kg/m^2^)	26.18 (22.95, 29.70)

Normal, n (%)	162 (40.3)

Overweight, n (%)	145 (36.1)

Obesity, n (%)	95 (23.6)

Presence/history of cerebrovascular accident, n (%)	17(4.2)

Positive history of hypertension, n (%)	219 (54.2)

**Diabetes mellitus, n (%)**	52 (12.9)

Heart rate at first presentation	

Bradycardia (< 60)	14 (3.5)

Normal (60–100)	267 (66.4)

Tachycardia (>100)	121 (30.1)

Reported Familial DCM n = 113	12 (10.6)


NI-DCM – Non-Ischemic Dilated Cardiomyopathy.

**Figure 1:**
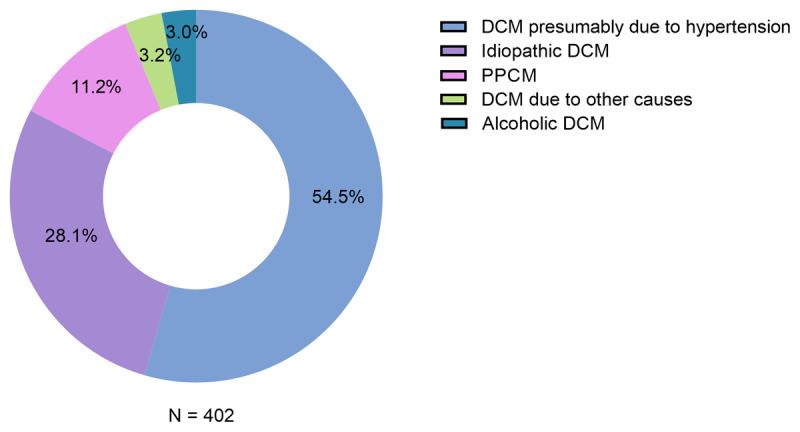
Causes of NIDCM in MOYO study.

Causes of NIDCM were presumably hypertensive n = 219 (54.4%), idiopathic n = 113 (28.1%), PPCM n = 45 (11.2%), alcoholic n = 12 (3.0%) and other causes n = 13 (3.2%), of which included infections (HIV and others), thyroid hormonal disease and chemotherapy-induced NIDCM, Figure 1.

**Figure 2:**
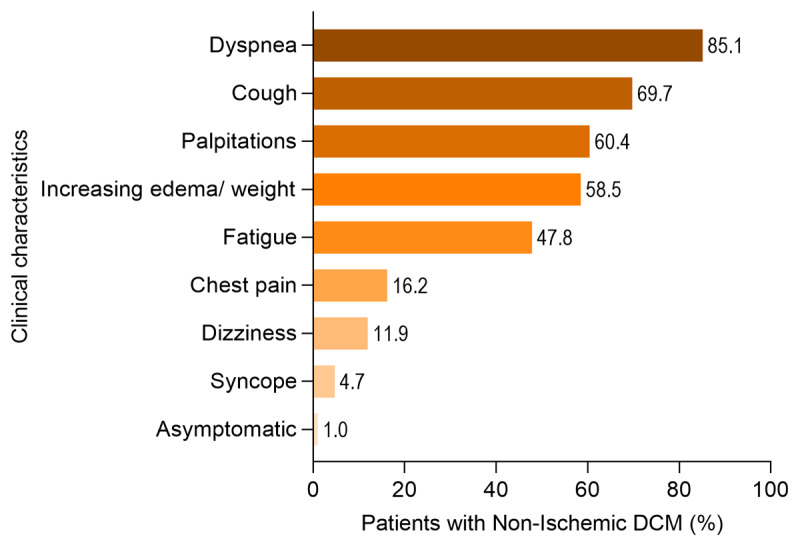
Clinical characteristics in NIDCM inMOYO study.

The most common presenting symptoms were dyspnea n = 342 (85.1%), followed by cough n = 280 (69.7%), palpitations n = 243 (60.4%), edema or increased weight n = 235 (58.2%) and paroxysmal nocturnal dyspnea n = 221 (55.0%), Figure 2.

Table 2 shows the echocardiographic findings, whereas the median (IQR) LV end-diastole diameter was recorded at 62.2 mm (58.6, 68.0). Mean (SD) LV ejection fraction was also markedly depressed at 29.4% (±7.7%), with n = 208 (51.7%) of patients presenting with severe LV systolic dysfunction defined as LVEF <30. Global RV systolic dysfunction was present in n = 145 (36.1%); assessment by TAPSE was performed in 342 patients with a median (IQR) of 16.0 mm (13.0, 18.0). Functional mitral n = 344 (85.5%) and tricuspid n = 266 (66.1%) valvular regurgitations were common. Intra-cardiac thrombi were seen in n = 25 (6.2%), mostly in the left ventricle n = 23 (5.7%).

**Table 2:** Echocardiographic findings in patients with NIDCM in Native Tanzanian Cohort: MOYO study.


VARIABLE	MEDIAN (IQR)/N (%)

LV end diastolic diameter (mm)	62.6 (58.6, 68.0)

LV dilation severity, n (%)	

Mild	52 (12.9)

Moderate	210 (52.2)

Severe	131 (32.6)

Unknown	9 (2.2)

Mean (SD) LV fractional shortening	14.7 (4.9)

Mean (SD) LV ejection fraction	29.45 (7.7)

LV dysfunction severity, n (%)	

Mild	39 (9.7)

Moderate	166 (41.3)

Severe	195 (48.5)

Unknown	2 (0.5)

RV dilatation, n (%)	240 (59.7)

RV dysfunction, n (%)	145 (36.1)

Median (IQR) TAPSE (mm) n = 342	16.0 (13.0, 18.0)

Mitral regurgitation, n (%)	344(85.5)

Mitral regurgitation severity, n (%)	

None or trace only	58 (14.4)

Mild	171 (42.5)

Moderate	87 (21.6)

Moderate to severe	7 (1.7)

Severe	79 (19.7)

Tricuspid regurgitation, n (%)	266(66.1)

Tricuspid regurgitation severity, n (%)	

None or trace only	136 (33.8)

Mild	127 (31.6)

Moderate	72 (17.9)

Moderate to severe	4 (1.0)

Severe	63 (15.7)

Aortic regurgitation, n (%)	53(13.1)

Aortic regurgitation severity n (%)	

None or trace only	349 (86.8)

Mild	38 (9.5)

Moderate	13 (3.2)

Moderate to severe	2 (0.5)

Intra-cardiac thrombosis, n (%)	25 (6.2)

LV severe systolic dysfunction	

LVEF <= 30 n (%)	

Yes	208 (51.7)

No	194 (48.3)


LV – left ventricle; RV – right ventricle; TAPSE – tricuspid annular plane systolic excursion; LVEF – left ventricular ejection fraction.

On chest radiographs (n = 273), the median (IQR) cardio-thoracic ratio was increased at 0.64 (0.59, 0.68), while cephalization, pulmonary edema, and pleural effusion were found in 44.0%, 32.2% and 30.4% respectively. On ECG, n = 331 (82.3%) had no bundle branch block, while left bundle branch block (LBBB) was seen in n = 60 (14.9%), premature ventricular contractions (PVC) were noted in 89 (22.1%) and atrial fibrillation was recorded in n = 44 (10.9%). Patients who underwent CAG (n = 36) had normal coronary arteries except one who had non-obstructive coronary disease, as shown in Table 3.

**Table 3:** Electrocardiography, Stress ECG, Coronary Angiography, Chest Radiography and Laboratory Findings in Patients with NIDCM in Native Tanzanian Cohort: MOYO Study.


VARIABLE	NUMBER STUDIED	MEDIAN (IQR)/N (%)

Median (IQR) Cardio-thoracic ratio	273	0.64 (0.59, 0.68)

Pulmonary edema, n (%)	273	88 (21.8)

Cephalization, n (%)	273	120 (29.8)

Pleural effusion, n (%)	273	83 (20.6)

Exercise stress testing	17	

ECG ST changes	17	0 (0.0)

ECG arrhythmias	17	7 (43.8)

CAG done yes n (%)	36	

CAG findings n (%)	36	

Normal coronary arteries		35(97.12)

Non-obstructive CAD		1(2.8)

CAG done as per cause of NIDCM	36	

NIDCM presumably due to hypertension		

Idiopathic DCM		21(60.0)

PPCM		12(34.2)

Alcoholic DCM		0(0)

NIDCM due to other causes		0(0)

Presence of bundle branch block	402	3(8.3)

No BBB		331 (82.3)

LBBB		60 (14.9)

RBBB		11 (2.7)

PVC seen Yes n (%)	402	89 (22.1)

Atrial fibrillation, yes n (%)	402	44 (10.9)

Median(IQR) serum Sodium (mmol/L)	150	134.0 (130.0,137.0)

Hyponatremia n (%)	150	99 (66.0)

Hypernatremia n (%)	150	1(0.7)

Anemia n (%)	226	127(56.2)


CAG – coronary angiograph; ECG – electrocardiograph; BBB – bundle branch block; LBBB – left bundle branch block; RBBB – right bundle branch block: PVC – premature ventricular contractions.

**Figure 3:**
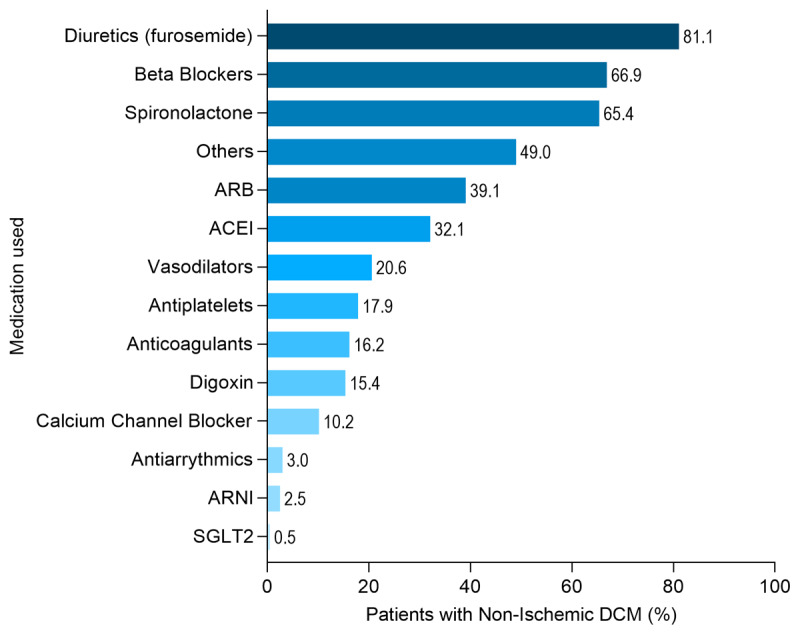
Medications used in NIDCM in MOYO study.

Beta-blockers, spironolactone, angiotensin II receptor blocker (ARB) and angiotensin converter enzyme inhibitor (ACEI) were used in n = 269 (69.9%), n = 263 (65.4%), n = 157 (39.1) and n = 129 (32.1), respectively. Meanwhile, angiotensin receptor-neprilysin inhibitor (ARNI) and SGLT2 inhibitors were least prescribed in n = 10 (2.5%) and n = 2 (0.5%), respectively, Figure 3.

In comparison to other etiologies of NIDCM, patients with idiopathic DCM were significantly younger, 50 years (40.0, 59.0) vs. 58.0 years (43.0, 68.0), p < 0.001; had more advanced NYHA class n = 30 (26.5%) vs. n = 37 (12.8%), p = 0.004 and more left bundle branch block in ECG n = 25 (22.1%) vs n = 35 (12.1%), p = 0.042, Table 4.

**Table 4:** Comparisons between patients with Idiopathic DCM and patients with other forms of NIDCM in characterization of NIDCM in native Tanzanian cohort: MOYO Study.


VARIABLE	IDIOPATHIC CAUSES OF DCM		p–VALUE

	YES n = 113 (%)	NO n = 289 (%)	

Median age in years (IQR)	50 (40, 59)	58 (43, 68)	<0.001

Age group <45			

Yes	42 (37.2)	78 (27.0)	0.045

No	71 (62.8)	211 (73.0)	

Sex			

Male	64 (56.6)	156 (54.0)	0.630

Female	49 (43.4)	133 (46.0)	

Medically insured			

Insured	52 (46.0)	162 (56.1)	0.070

Not insured	61 (54.0)	127 (43.9)	

NYHA class at presentation			

I	3 (2.7)	5 (1.7)	0.004

II	37 (32.7)	95 (32.9)	

III	43 (38.1)	152 (52.6)	

IV	30 (26.5)	37 (12.8)	

Familial DCM			

Yes	12 (10.6)	25 (8.7)	0.539

No	101 (89.4)	264 (91.3)	

Median LVEF (IQR)	28.7 (22.1, 32.6)	29.5 (23.6, 37.0)	0.073

LVEF category <30			

Yes	62 (54.9)	146 (50.5)	0.433

No	51 (45.1)	143 (49.5)	

Presence of bundle branch block			

No BBB	86 (76.1)	245 (84.8)	0.042

LBBB	25 (22.1)	35 (12.1)	

RBBB	2 (1.8)	9 (3.1)	

PVC seen			

Yes	29 (25.7)	60 (20.8)	0.295

No	84 (74.3)	228 (79.2)	

Atrial fibrillation			

Yes	8 (7.1)	36 (12.5)	0.118

No	105 (92.9)	252 (87.5)	


BBB – bundle branch block; LBBB – left bundle branch block; RBBB – right bundle branch block; PVC – premature ventricular contractions.

## Discussion

Characterization of non-ischemic dilated cardioMyOpathY in a native Tanzanian cOhort is the first study performed in Tanzania to characterize patients with NIDCM specifically. The study was initiated to gain insights into the similarities and differences between different NIDCM cohorts regionally as well as globally.

The median age of our cohort is lower compared to published data from the PARADIGM-HF trial. However, in the PARADIGM-HF trial, age was recorded according to the investigator-reported cause of heart failure, where mean ages for hypertensive, idiopathic and other causes of heart failure were 64.7, 60.0 and 58.3 years, respectively. Conversely, males’ predominance is more pronounced in PARADIGM-HF than in our cohort, while interestingly, non-ischemic idiopathic patients in PARADIGM-HF were more frequently female and Asian [25].

Our series contrasts with registries in Europe, where most patients have a higher level of education and robust health insurance, which potentially translates into a better understanding of their condition and compliance with management [26]. However, our findings are similar to the INTERnational Congestive Heart Failure (INTER CHF) study which enrolled patients with heart failures in 16 countries, among which 1294 patients were from Africa. African participants were younger, had lower literacy levels, and were less likely to have health insurance [27].

### Cardiovascular risk factors in patients with NIDCM

Non-ischemic dilated cardiomyopathy, presumably due to hypertension in our cohort, is generally higher than observed in the PARADIGM-HF trial. The trial involved patients from Europe, Asia and Latin America; hypertensive etiology was most common in Latin America (21% of all cases) and least common in the Asia-Pacific [25]. The difference may thus be explained by ethnicity since black ethnicity, predominant in our cohort, is an important risk factor for hypertension [28]. Also, other factors need to be considered in explaining this phenomenon, such as the lack of hypertension screening programs in Tanzania.

Our study recorded comparable findings to a study in Sudan, where alcohol abuse was seen in 14.6% of the patients with DCM [29]. A case-control study to compare the prevalence of nine genes associated with inherited DCM noted that DCM-causing genes were more prevalent in patients with alcoholic cardiomyopathy than in control subjects [30].

A study by Rayner et al. reported a relationship between increasing BMI and left ventricular (LV) remodeling in patients with NIDCM (n = 529). It recorded a difference in the degree of LV cavity dilatation associated with change in stroke volume; when compared to normal hearts [increase in end-diastolic volume of 0.7 mL per unit of rising BMI (mL/kg/m^2^)], there was a threefold greater LV cavity dilatation in NIDCM (+2.2 mL/kg/m^2^) [31]. Almost a quarter of participants in our series were obese, increasing their chances of developing LV remodeling as a complication, which may be compounded by other existing risk factors leading to NIDCM.

Hyperglycemia seen in patients with diabetes mellitus type 2 (T2DM) has been associated with cardiovascular alterations such as endothelial dysfunction, adverse effects of circulating free fatty acids (FFA) and increased systemic inflammation [32]. Another study which compared NIDCM (T2DM–) and NIDCM (T2DM+) patients reported increased LV end-diastolic and end-systolic volume index and decreased LV ejection fraction. LV global strains progressively declined from the normal controls to the NIDCM (T2DM–) group to the NIDCM (T2DM+) group (all p < 0.017). In that study, NIDCM patients with T2DM showed an association between HbA1c and reduced LV myocardial strain on Cardiac MRI [33]. In our series, we had some cases of T2DM; we need further studies with a larger sample to elucidate the role of hyperglycemia in NIDCM in Tanzania.

Cigarette smoking increases inflammation and triggers fibrotic processes, including cardiac fibrosis; this has a strong correlation with systolic dysfunction, diastolic dysfunction and abnormal electrical excitation [34]. A study done in South Korea involving NIDCM patients using Cardiac MRI concluded that smokers had higher fatal ventricular arrhythmic events and poorer outcomes compared to non-smokers [34]. There is a possibility that most patients in our study did not report smoking. However, they could be consuming other forms of tobacco, such as snuff, which may be detrimental to the cardiovascular system.

### Idiopathic DCM

The age of disease manifestation in our series concurs with current literature; it has been proven that most cases of Idiopathic DCM have a genetic origin. A meta-analysis involving 8,097 patients with idiopathic DCM in 40 studies showed that the average frequency of mutations in the investigated genes was between 1 and 5%. The mean age of idiopathic DCM onset was the beginning of the fifth decade for all genes [35]. This finding brings attention to the importance of genetic screening in this subgroup in the current era of precision medicine.

Similar to our series, Bailly et al. analyzed 50 pedigrees with idiopathic DCM and found that 14 (28%) exhibited signs indicative of familial DCM dilated cardiomyopathy [36]. Idiopathic DCM typically has a protracted asymptomatic phase, so its true frequency of familial tendency can only be fully understood through comprehensive family screenings. A subset of the MOYO cohort was subjected to a family screening (n = 120); it was revealed that 17 individuals (14.7%) had familial disease [37]. This observation underscores the importance of implementing routine family screening to comprehend the extent of the problem.

### Peripartum dilated cardiomyopathy (PPCM)

The proportion of patients with PPCM in our study is comparable to other studies in Africa. A Nigerian study showed that PPCM was the predominant form of cardiomyopathy in females found in 31.4% [38]. Conversely, the proportion of PCCM in our study is high when compared to other regions, such as Europe. A retrospective study done in Denmark recorded an incidence of one in 10,149 deliveries [39]. These findings call for better management of peripartum health in African women, as it has been noted that Black women are at higher risk of developing PPCM [40].

### NIDCM of other causes

Some of our patients presented with underlying viral infections such as HIV; the number of patients with HIV-induced cardiomyopathy seems smaller in this era where patients are on highly active antiretroviral therapy (HAART) regimens compared to pre-HAART era [41]. Pathogenesis of NIDCM in patients with viral infection is explained by different etiological triggers confluence into a common autoimmune process leading to chronic inflammation, tissue remodeling and fibrosis [41].

### Clinical symptoms and disease severity

Our findings differ from the European population; palpitations were only recorded in 36% of cases, and most patients presented with NYHA II at 42.7%, while in our series, the majority presented with more clinically advanced disease [42]. The disparity among these cohorts can be explained by better awareness and access to care in European countries and existing screening programs.

### Echocardiographic and chest radiography findings

The majority of our patients presented with low LVEF, which implies severe systolic dysfunction and mitral valve regurgitation, which may be explained by an advanced state of the disease. A multi-centric international study observed that LVEF was an independent predictor of all-cause mortality [43].

Increased cardiothoracic ratio (CTR) in most patients in this cohort is consistent with a study done in South Africa, whereby increased CTR was seen in patients with DCM. Radiographic findings were confirmed by echocardiography as increased LV end-diastolic diameter [44]. Pulmonary edema seen in our patients is comparable to other studies in Africa [29].

### Management of patients with DCM

Recently published data from the European Registry of Cardiomyopathy and Myocarditis (EORP) identifies beta-blockers as the pillar of management, as seen prescribed in 90% of their cases, followed by ACEIs or ARBs at 89% [42]. These findings contrast with our setting, where diuretics were the most commonly prescribed due to advanced disease; however, there is a remarkable use of beta-blockers and limited use of ARNI and SGLT2 inhibitors. The unavailability of medication, financial constraints for most cases and lack of insurance coverage for novel drugs may explain the limited use.

### Limitations/mitigation

In this study, a number of challenges were observed, including limited coverage of medical insurance and limited numbers of diagnostic facilities with high patient load. These limitations led to challenges in the investigations of patients as per internationally established protocols. Echocardiography was used as the main diagnostic tool for most patients. A significant number of patients lacked routine laboratory tests in their records. However, JKCI is the only specialized institute in the country; the data in this study provides insight into the Tanzanian population as far as NIDCM diagnosis and care status, thus guiding us into a better understanding of the condition as well as establishing appropriate management guidelines.

## Conclusion

The MOYO study has demonstrated the feasibility of prospective characterization of a subset of patients with heart failure due to NIDCM in Tanzania. The findings of this study may enlighten health policies in forming tailored cardiovascular care for such a complex cohort. Notably, it has been found that patients with idiopathic DCM were often young, more often male, with no formal to a low level of education when compared to the main international registries. While most cases were due to idiopathic or presumed hypertensive cardiomyopathy, more than one in every ten patients had PPCM. Furthermore, at least half of the patients presented with severe heart failure, with the majority in NHYA functional class III–IV and LVEF <30, yet guideline-directed medical therapy was suboptimal. These findings are a call to action, especially the need for an earlier diagnosis and strategies to facilitate evidence-based, proven treatments.
